# Valedictory Address

**Published:** 1871-03

**Authors:** Henry Cabell Jones


AMERICAN JOURNAL

OF

### DENTAL SCIENCE.

Vol. IV. THIRD SERIES--MARCH, 1871. No. 11.

### ARTICLE I.

### *Valedictory Address.*

Delivered at the Thirty-first Annual Commencement of the Baltimore College of Dental Surgery, by Henry Cabell Jones, D.D.S., of the Graduating Class.

Ladies and Gentlemen:—Conforming to a custom, the present graduating class of the Baltimore College of Dental Surgery have selected one of their number to represent them in their commencement exercises, and I have the honor to address you in that capacity.

Many years will have to run their course, ere the remembrance of the earnestness of your good wishes and approval, manifested by your presence here to-night, and the oft repeated kindly attentions which we have received at your hands, will be erased from the tablets of our memories. To-night we meet together for the last time, for it is not in the course of human events that we shall ever meet again, and our parting will be one in which sad and joyous emotions will be so intimately blended, that it will be difficult to strike the balance between them. Painful indeed will it be to sever the fond associations which have entwined themselves around us; sad indeed to part from the many friends made during our sojourn in this beautiful city. But joyou in another view will be this parting, for in a few shor hours we will have arrived at our homes, have been received by the loved ones who have fondly hoped and earnestly prayed for our safe return, and as a recompense for our separation, we will be able to place in their hands the certificate of having performed our duty this winter. We can say with honest pride that we have received our passports into active professional life, and may indulge the trust that with their assistance, we will never cease our efforts to attain that skill in the practice of our profession which will enable us to command the confidence of the communities in which our several lots shall be cast.

Dear friends, we truly appreciate the many evidences of the earnestness of your good wishes and approval manifested here to-night, and we would one and all extend to you the warm hand of friendship, feeling sincerely thankful for the heartiness of your encouragement.

*Respected Gentlemen of the Faculty:*—Words cannot express our gratitude to you for the faithful, untiring manner in which you have instructed us in the fundamental truths of our profession, for the principles which you have instilled into our minds, for the kind forbearance with which you have borne with us, for the words of encouragement which you have spoken; when well nigh discouraged, almost despairing, you have bidden us hope. Never will we forget with what cordiality you have at all times received us, treating us rather as sons than as students. As a reward for our past study, we have to-night received at your hands our diplomas as Doctors of Dental Surgery, and now as we bid adieu to the lecture room and pass out into active life, our strongest bond of obligation is so to acquit ourselves as to receive your approval. We crave no higher boon, than that you may never regret the confidence which your have this night reposed in us, and that you may look with pride upon our prosperity and success. The relations existing between teacher and pupil, akin to that of father and son are amongst the most lasting in life. The gloom of sadness settles upon our brows when we realize that these are our parting words, that associations so pleasant and profitable are to be severed, and we would not leave you without making known our thorough appreciation of your efforts, nor have you remain ignorant of the lasting attachments which we formed for you individually.

Wherever in the wide world our lot may be cast, and whatever fortune betide, the seeds of truth, good motives and sound principles, which you have so carefully sown in our minds, we trust will germinate and bring forth such fruit as will obtain for us the prize in all contests. May a long life be your portion, may many more be entrusted to your hands for instruction, may you reap year after year the abundant rewards of the faithful laborers, and when the noontide of life is past, end your days in peace plenty and happiness is our most earnest prayer.

And now my *Fellow Classmates*, to you it is meet I should address a few remarks. To-night we, in receiving our diplomas, have that guarantee which admits us to honorable competition with those who have already acquired eminence in the ranks of professional life. Let us ever maintain that self-respect and high moral standard which our professsion demands of us; let us beware of all vices, for whoever has a vice has a vulnerable place in the wall of his moral defence; he may be strong at all other points, but though elsewhere locked in triple steel, he is there open to successful attack. The weaknesses of character are the least guarded; the one of vulnerable character feels strongest in that point where he is most open to attack. The suspicious and jealous pretend unusual confidence. The rash and impetuous boast of discrimination and coolness. Cautions are wasted, for vanity makes them deaf and blind; self confidence is not safety, except for those who trust to what is sound, tried, solid, true. Vices grow weak by numbers; virtues stand in serried rank, strong, disciplined, and mutually strengthening; vices are a loose, disorderly, contentious throng, each making the other worse and all combining against the possessor. He whose passions, of whatever kind, overcome his reason, will see his vices in the garb of virtues. Whether his ruling vice be ambition or avarice, vanity or cruelty, inconstancy or fear, dishonesty, selfishness or procrastination, when he comes to take a calm and sorrowful review of his life's history, he will be able to trace most of his suffering, the worst of his failures, the most serious of his blunders, to his leading vice, or the combined influence of his several vices. He will then see clearly, what he may have dimly seen or only suspected before, that a vice is a defeat of moral vision, distorting all persons, objects and facts. He will feel what precepts could never teach him, that vice takes the senses captive. Let us therefore study closely our characters and wherever we find a vice, tear it up by the roots and plant in its stead a virtue, to present an unbroken phalanx, which our enemies will be unable to penetrate. With a front thus watched and fully guarded, we will be able to attain that enviable place in the opinions of our fellow men, which will justify them in saying of us, he is a true man.

"Resolve is what makes a man manliest, not puny resolve, not crude determination, not errant purpose, but that strong and indefatigable will which treads down difficulties and danger, as a boy treads down the heaving frost-land of winter, which kindles his eye and brain with a proud pulse-beat toward the unattainable. Will makes men giants. It made Napoleon emperor of kings; Bacon a fathomer of nature; Byron a tutor of passion, and the martyrs masters of death;" it will make us masters of our profession. Then fellow classmates let us resolve, and resolving perform, that no difficulty however great, no barrier however insurmountable, shall make us falter; but ever with the determination to trample under foot all difficulties, to conquer all barriers, let us walk our way in life, that when our pilgrimage here on earth is ended and the summons to eternity is received, each of us can say truly, I have performed my duty to my God and to my fellow men.

In that supreme hour no reflection will afford us any genuine confidence, any more satisfaction, but this. No success, no public applause, no private aggrandizement will disarm that moment of the most trifling of its terrors, and yet all that clothes it with its most appalling gloom, will disperse as mists before summer suns under the single reflection, "I have done my duty."

> "So live that when thy summons come to join
> The innumerable caravan, that moves
> To the pale realms of shade, where each shall take
> His chamber in the silent halls of death;
> Thou go not, like the quarry-slave at night,
> Scourged to his dungeon; but sustained and soothed
> By an unfaltering trust, approach the grave,
> Like one who wraps the drapery of his couch
> About him, and lies down to pleasant dreams."

That this may be the happiness of each of you and of all this audience, is the highest blessing I can invoke as I say "farewell."

